# Deficient Response to Experimentally Induced Alkalosis in Mice with the Inactivated insrr Gene 

**Published:** 2011

**Authors:** I.E. Deyev, D.I. Rzhevsky, A.A. Berchatova, O.V. Serova, N.V. Popova, A.N. Murashev, A.G. Petrenko

**Affiliations:** Shemyakin and Ovchinnikov Institute of Bioorganic Chemistry, Russian Academy of Sciences; Branch of Shemyakin and Ovchinnikov Institute of Bioorganic Chemistry, Pushchino, Russian Academy of Sciences

**Keywords:** alkalosis, IRR

## Abstract

Currently, the molecular mechanisms of the acid-base equilibrium maintenance in the body remain poorly understood. The development of alkalosis under various pathological conditions poses an immediate threat to human life. Understanding the physiological mechanisms of alkalosis compensation may stimulate the development of new therapeutic approaches and new drugs for treatment. It was previously shown that the orphan insulin receptor-related receptor (IRR) is activated by mildly alkaline media. In this study, we analyzed mutant mice with targeted inactivation of the*insrr *gene encoding IRR, and revealed their phenotype related to disorders of the acid-base equilibrium. Higher concentrations of bicarbonate and CO_2_were found in the blood of*insrr *knockout mice in response to metabolic alkalosis.

## INTRODUCTION

The insulin receptor-related receptor (IRR) is a receptor tyrosine kinase that belongs to the minifamily of the insulin receptor, which also includes the insulin receptor and insulin-like growth factor receptor [1]. The cDNA sequence of IRR was cloned in 1989 [2]; however, as of now no natural agonists for IRR possessing a peptide or protein character have been found [3].

Contrary to its close homologs, which are present in a large number of tissues and cells, IRR is produced only in some tissues and specific cell populations. The largest concentration of IRR is found in the kidneys, where this receptor is present only in β-intercalated cells (a subpopulation of epithelial cells lining the distal ducts) [4]. These cells are in contact with the renal filtrate, the pH of which, in contrast to blood, may vary to become alkaline. IRR is also expressed in gastric enterochromaffin-like cells [5] secreting histamine, which, in turn, stimulates the secretion of acid, accompanied by an outflow of alkali from the stomach wall to the blood stream. A significant amount of IRR was detected in β- and α-cells of the islets of Langerhans, which may be in contact with the alkaline pancreatic juice [6]. It was found that IRR, in contrast to its homologs, can be activated at pH > 8.0 [7, 8] and presumably is a cellular sensor of mildly alkaline media. The postulated function of IRR correlates well with its known tissue and cell distribution.

**Fig. 1:**
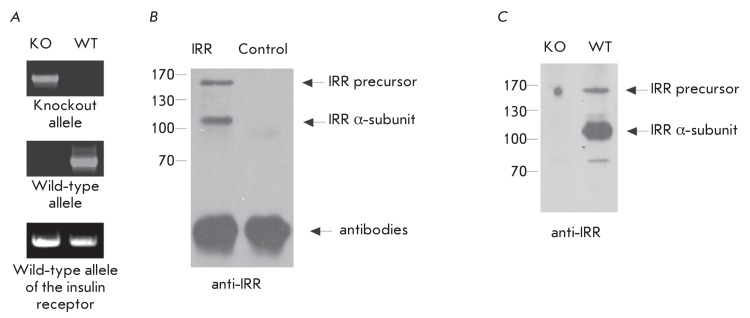
Analysis of the inactivation of the *insrr * gene in mice. ( *А* ) – PCR on the genomic DNA of the wild-type and *insrr * knockout mice with the use of the primers to the wild-type allele of IRR, IR and the *insrr* knockout allele. ( *B* ) – Western blotting with the use of antibodies against the IRR ectodomain. Lysates from transfected HEK293 cells with IRR-HA expressing plasmid and non-transfected cells (as the negative control) were blotted with the anti-ectodomain IRR antibodies. ( *C* ) – Equal amounts of WGA-eluates (about 10 µg) from the kidney membrane fraction of wild-type and *insrr* knockout mice were blotted with anti-ectodomain antibodies.

In order to reveal the functional properties of IRR, we performed a phenotypic analysis of the mice with the genetically ablated *insrr * gene that encodes IRR. It was found that the *insrr * knockout mice had a defect in the compensatory response to experimentally induced alkalosis. 

## EXPERIMENTAL

**Antibodies and Western-Blot Analysis**

 We used the rabbit antibody against the 539–686 mouse IRR fragment fused with glutathione S-transferase (GST) [8]. Monospecific antibodies were purified on a matrix with the 539–686 mouse IRR fragment and a 6-His tag coupled with BrCN-Sepharose. The proteins were separated by electrophoresis on a 10% polyacrylamide gel (PAAG) in the presence of sodium dodecyl sulfate (SDS) and were subsequently transferred to a nitrocellulose membrane. In order to reduce nonspecific sorption, the membrane was incubated in a milk/TBS-T buffer (5% non-fat milk, 20 mM Tris-HCl, pH 7.6, 140 mM NaCl, and 0.1% Tween-20) overnight at a temperature of 4°C. In order to detect the proteins, the membrane was incubated with the primary antibodies (1 : 5000 by volume) for 60 min at room temperature, washed with TBS-T, and then incubated with the secondary anti-rabbit antibodies conjugated with horseradish peroxidase (1 : 10000 by volume) for 1 h. The bound antibodies were detected by SuperSignal West Pico chemiluminescent substrate [9]. 

**Animal Experiments**

 The *insrr * knockout mice were obtained by *in vitro* fertilization with frozen sperm of the insulin receptor family triple knockout mice [10]. The wakeful, sexually mature male mice with targeted inactivation of only the *insrr * gene were examined. Genotyping was performed in accordance with [10]. The C57BL6 mice were taken as a control group. The animals were kept in the Laboratory Animal Breeding Facility (Branch of the Institute of Bioorganic Chemistry, Pushchino) under ambient conditions (temperature of 21 ± 2°C, humidity of 30–70%, and a lighting cycle of 12/12 h), mice were given *ad libitum* access to food and water. 

The experiments were performed upon wakeful animals. A 1.3% solution of NaHCO _3_ (200 µl/10 g of body weight) was injected into the tail vein of mice within 5 s. The blood samples were taken 30 min prior to the injection of NaHCO _3_ (point 0), as well as 5 and 15 min after the injection. Blood was collected from the retro-orbital sinus into plastic capillaries (Lithin-Heparin, 50 unit/ml) and analyzed with the help of a Rapidpoint 405 blood gas analyzer (Siemens). All manipulations were performed in accordance with the protocol certified by the Institute Committee on the Control of the Housing and Use of Laboratory Animals. 

## RESULTS

**Table 1:** Blood parameters in wild-type (WT) and *insrr * knockout mice (KO)

Electrolytes in blood	WT mice		KO mice	
	Mean value	Error	Mean value	Error
рН	7.21	0.03	7.29	0.02
РСО_2_, mm Hg	50	1.5	49	1.5
РО_2_, mm Hg	41	0.8	42	0.8
BE, mmol/l	-8.4	1.8	-4.2	1.2
TCO_2_, mmol/l	21.4	1.6	24.4	1.0
HCO_3_,mmol/l	19.9	1.6	22.9	1.0
Na, mmol/l	148	0.9	148	0.8
K, mmol/l	6.2	0.1	6.3	0.2
Ca, mmol/l	1.25	0.0	1.24	0.0
tHb, g/l	16.8	0.3	18.0	0.4
Hct, %	50	1.0	54	1.1

**Fig. 2:**
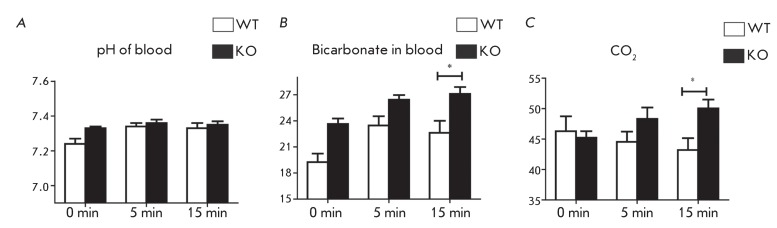
Blood gas analysis of blood from wild-type (WT) and *insrr* knockout (KO) mice before and after injection of a bicarbonate solution. ( *А* ) – рН of blood; ( *B* ) – concentration of bicarbonate in blood (mmol/l); ( *C* ) – concentration of СО _2_ in blood (mm Hg). * *p*  < 0.05 by the Student’s test.

In order to obtain a homozygous line of the *insrr* knockout mice, animals with the *insrr * gene, as well as those with the insulin receptor and insulin-like growth factor receptor genes knocked out (triple heterozygous knockout [10]), were crossed with the C57BL6 mice of the wild type. The presence of alleles with the *insrr * gene knocked out and the absence of alleles with the insulin receptor and insulin-like growth factor receptor genes knocked out were verified by PCR on the genomic DNA of mice ( *Fig. 1A* ). 

The synthesis of IRR protein in mice with the *insrr * gene knocked out was analyzed using the antibody that specifically recognizes the ectodomain of IRR ( *Fig. 1B* ). The Western blots of partially purified renal membrane extracts of the normal mice and the *insrr* knockout mice were stained with these antibodies. This analysis confirmed that no IRR protein was present in the obtained mice ( *Fig. 1C* ). 

The primary analysis performed under normal conditions did not reveal any significant differences between the normal mice and the mice with the *insrr* gene knockout [11]; hence, we performed two series of experiments with the wild-type and knockout mice both under normal conditions, as well as under induced alkalosis. 

In the first series of experiments, we determined 11 blood parameters in mice from both groups (eight animals per group) under normal conditions. It was found that in mice with the inactivated *insrr * gene, the concentration of bicarbonate in blood (22.9 ± 1.0 against 19.9 ± 1.6, *p*  < 0.05), as well as pH (7.29 ± 0.02 against 7.21 ± 0.03, *p*  < 0.05), and the hematocrit (54 ± 1.1 against 50 ± 1.0, *p*  < 0.05) was higher than that in the wild-type animals. All other determined blood parameters did not differ significantly ( *Table* ). 

For the second series of experiments, two groups of animals were selected: 10 wild-type mice and 12 knockout mice. Metabolic alkalosis was induced by an intravenous injection of a 1.3% NaHCO _3_ solution (200 µl/10 g of body weight). The blood parameters were determined at intervals of 5 and 15 min following the alkaline injection. Five minutes after the injection of the alkaline solution, the dynamics of the variation in the concentration of bicarbonate and blood pH in mice with IRR knocked out was the same as that in the wild-type mice; i.e., pH increased (from 7.24 ± 0.03 to 7.34 ± 0.02, *p * < 0.05 in wild-type mice and from 7.33 ± 0.01 to 7.36 ± 0.02, *p*  < 0.2 in knockout mice) and so did the concentration of bicarbonate (from 19.25 ± 0.98 to 23.47 ± 1.06, *p*  < 0.05 in wild-type mice and from 23.64 ± 0.63 to 26.43 ± 0.53, * p*  < 0.05) ( *Figs. 2A, B* ). Fifteen minutes after the induction of alkalosis, the blood pH value in mice from both groups became somewhat lower than that recorded after 5 min (7.33 ± 0.03 in wild-type mice and 7.35 ± 0.02 in knockout mice). However, after 5 min, a significant increase was observed both in the concentrations of bicarbonate and CO _2_ in the blood of the mice with IRR knocked out in comparison with these values in the wild-type mice ( *Figs. 2B, C* ); thus, the wild-type animals and the animals with the *insrr * gene knockout displayed different responses to acute alkalosis, which was experimentally induced via the injection of bicarbonate into the blood. 

## CONCLUSIONS

It was demonstrated in our previous studies that IRR is a sensor of extracellular alkaline media. The absence of this gene in the body leads to a disturbance in the compensation of metabolic alkalosis, induced by feeding animals with alkaline food for several days. This effect resulted from a defect in bicarbonate secretion by the kidneys of the *insrr* knockout mice [8]. The results obtained in studying the compensation of induced alkalosis under acute conditions (5–15 min) confirm our hypothesis on the compensatory role of IRR in the secretion of bicarbonate. It can be concluded that IRR-dependent compensation of alkalosis proceeds rather fast. It is important to note that, in mice with IRR knocked out, compensation of alkalosis is also observed (since the blood pH value decreases). However, this occurs not due to the secretion of excess amounts of bicarbonate, but as a result of the increase in the concentration of CO _2_ in the blood; the latter most likely results from the decelerated breathing or accelerated metabolism. Thus, it can be concluded that IRR plays a significant role in the physiological mechanisms of regulation of the acid-base equilibrium and that mice with IRR knocked out can be used as animal models to study pathological development of metabolic alkalosis. 
